# Chromosome-level phased genome assembly of the argan tree *Sideroxylon spinosum*

**DOI:** 10.1038/s41597-025-05768-1

**Published:** 2025-08-15

**Authors:** Ivan D. Mateus, Abdellatif Essahibi, Pamela Nicholson, Mohamed Hijri, Ahmed Qaddoury, Laurent Falquet, Didier Reinhardt

**Affiliations:** 1 Department of Biology, University of Fribourg, Chemin du Musée 10, 1700 Fribourg, Switzerland; 2 Next Generation Sequencing Platform, University of Bern, Bremgartenstrasse 109a, 3012 Bern, Switzerland; 3 African Genome Center, University Mohammed VI Polytechnic (UM6P), Lot 660, Hay Moulay Rachid, 43150 Ben Guerir, Morocco; 4 Department of Biology, Faculty of Sciences and Techniques, University of Cadi Ayyad, Marrakesh, Morocco; 5 Swiss Institute of Bioinformatics, Lausanne, Switzerland

**Keywords:** Eukaryote, Classification and taxonomy

## Abstract

Argan (*Sideroxylon spinosum* L., formerly *Argania spinosa*) is a tree endemic to Morocco, primarily valued for its seed oil. Growing interest in its biology and in genes linked to oil quality and stress resistance highlights the need for high-quality genome and transcriptome models. We integrated PacBio HiFi long-read and Illumina Hi-C sequencing data to generate independently assembled, phased genome models for both parental haplotypes, measuring 636 Mb and 655 Mb, respectively, with BUSCO completeness scores exceeding 97.8%. Each haplotype consists of 11 fully resolved telomere-to-telomere chromosomes, consistent with chromosome numbers in other Sapotaceae species (n = 10–13), and contains approximately 60% repetitive sequences. Annotation predicted ~28,720 protein-coding genes per haplotype. Comparative analyses with other Sapotaceae genomes indicate overall chromosome conservation within the family, alongside repeat expansion and fusion events in the two largest chromosomes (chr1 and chr2). We also independently assembled the complete chloroplast genome. This high-quality assembly provides a valuable resource for future research on argan biology, genetic diversity, and traits relevant to adaptation and oil biosynthesis.

## Background & Summary

Argan is a member of the Sapotaceae family (order Ericales), which comprises five tribes and approximately 1,250 species. This family includes economically important species valued for their oil, such as the argan tree (*Sideroxylon spinosum* L., formerly *Argania spinosa* (L.) Skeels), the shea tree (*Vitellaria paradoxa* C.F. Gaertn.), and the miracle fruit tree (*Synsepalum dulcificum* (Schumach. & Thonn.) Daniell). Argan seeds contain an edible oil extensively used for both culinary and cosmetic applications. Argan oil represents a crucial economic resource for many Moroccan families^1^, particularly for women, who play a central role along the entire value chain. Argan is a diploid species with a reported haploid chromosome number ranging between 10 and 12^2^.

Argan has not been selectively bred or extensively cultivated, instead, it grows in its natural habitat, where it has been sustainably utilized for centuries. Nevertheless, overgrazing by goats, the intensification of land use in Morocco, and the high demand for argan oil have led to unsustainable exploitation, putting the species at risk^1^. In recent years, rising temperatures, prolonged droughts, and an extended dry season have further exacerbated the challenges for the argan-growing region, known as the “Arganeraie”, which has been listed as UNESCO biosphere heritage in 1998.

The economic importance of argan has raised the demand for high-quality genomic resources. A first genome draft containing 75,327 scaffolds^3^, was followed by a reference annotation with 62,590 genes^4^. The complete mitochondrial and chloroplast genomes have also been released in recent years^5,6^. Additionally, single-sequence repeat markers^7^ and AFLP markers^8^ have been developed for the use in population genetics studies, revealing high genetic diversity between populations but low diversity within populations^7–9^. However, a high-quality genome sequence is required for a more comprehensive understanding of argan evolution, physiology, population genomics, as well as for marker-assisted breeding.

In this study, we used PacBio HiFi sequencing combined with Illumina Hi-C to generate a complete genome assembly of the argan tree with two independently assembled haplotigs and the chloroplast genome. We compared our phased assembly with genome assemblies of two other Sapotaceae species to investigate chromosome composition and evolution. Our telomere-to-telomere genome assembly provides a high-quality reference for comparative genomics, offering new insights into chromosome evolution in this species.

## Methods

### Sample collection and sequencing

Leaf samples of *Sideroxylon spinosum* (syn. *Argania spinosa*) were collected from a specimen at the northern border of the UNESCO biosphere reserve “l′Arganeraie”, along the National Road 207 east of Essaouira at coordinates 31°32′47.9″ N 9°21′56.6″ W (Fig. S1). We employed a hybrid sequencing strategy that combined PacBio long-read sequencing with Hi-C chromatin conformation data. Genomic DNA was extracted from leaves using a modified CTAB protocol (**Supplementary Methods**). For Hi-C sequencing, nuclei were isolated using a Sucrose/Percoll gradient centrifugation protocol (**Supplementary Methods**). To generate highly accurate long reads, we performed Pacific Biosciences (PacBio) Sequel II HiFi sequencing on sheared genomic DNA (gDNA). Hi-C sequencing was carried out using Illumina NovaSeq 6000 (paired-end, 150 bp) on crosslinked chromatin, which was enzymatically digested with DpnII (^GATC) and HinfI (G^ANTC) using the Arima Genomics Hi-C kit (Cat. #A510008), followed by proximity ligation to capture three-dimensional chromosomal interactions.

### Genome assembly

**Fig. 1:**
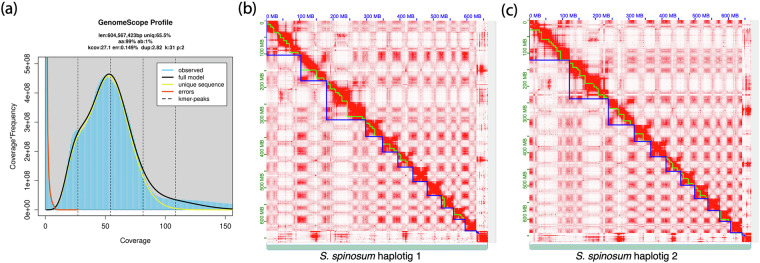
Global genome characterization for *S. spinosum*. (**a**) GenomeScope genome size estimates for *S. spinosum*. (**b,c**) Genome-wide chromosomal Hi-C maps of haplotig 1 (**b**) and haplotig 2 (**c**) of *S. spinosum*; chromosomes are delineated in blue, and contigs are highlighted with a green outline.

The genome of *S. spinosum* was assembled by integrating long-read PacBio HiFi sequencing with Hi-C data to establish physical linkage. High sequence coverage allowed for the independent assembly of the two parental genomes, each consisting of 11 chromosomes. PacBio HiFi sequencing reads were quality-checked using FastQC v.0.11.9 and cleaned with fastp v.0.19.5^10^. The cleaned HiFi reads were assembled into phased haplotigs using hifiasm v.0.16.1 with –hom-cov 50 parameters^11^. K-mer analysis performed with GenomeScope 2.0 (http://genomescope.org)^12^ revealed a low error rate and a high homozygosity between the two haplotigs (Fig. 1a). The haplotigs were further scaffolded using Illumina Hi-C data. We applied HiCup to map the Hi-C reads to the hifiasm assemblies^13^, and we used Juicer v1.6^14^ and YAHS v1.2.2^15^ to generate the final telomere-to-telomere (T2T) chromosome assemblies. The YAHS file “telo.c” was customized to include the telomeric sequences of *S. spinosum* TTTAGGG & GGGTGGG and the software was recompiled before generating the final T2T chromosome assemblies. The final assembly was manually curated with JuiceBox (v2.15)^16^ (Fig. 1b,c). The telomeric regions were identified using tidk (v0.2.31)^17^ with the canonical telomeric sequences TTTAGGG and GGGTGGG (Fig. S2).

**Fig. 2:**
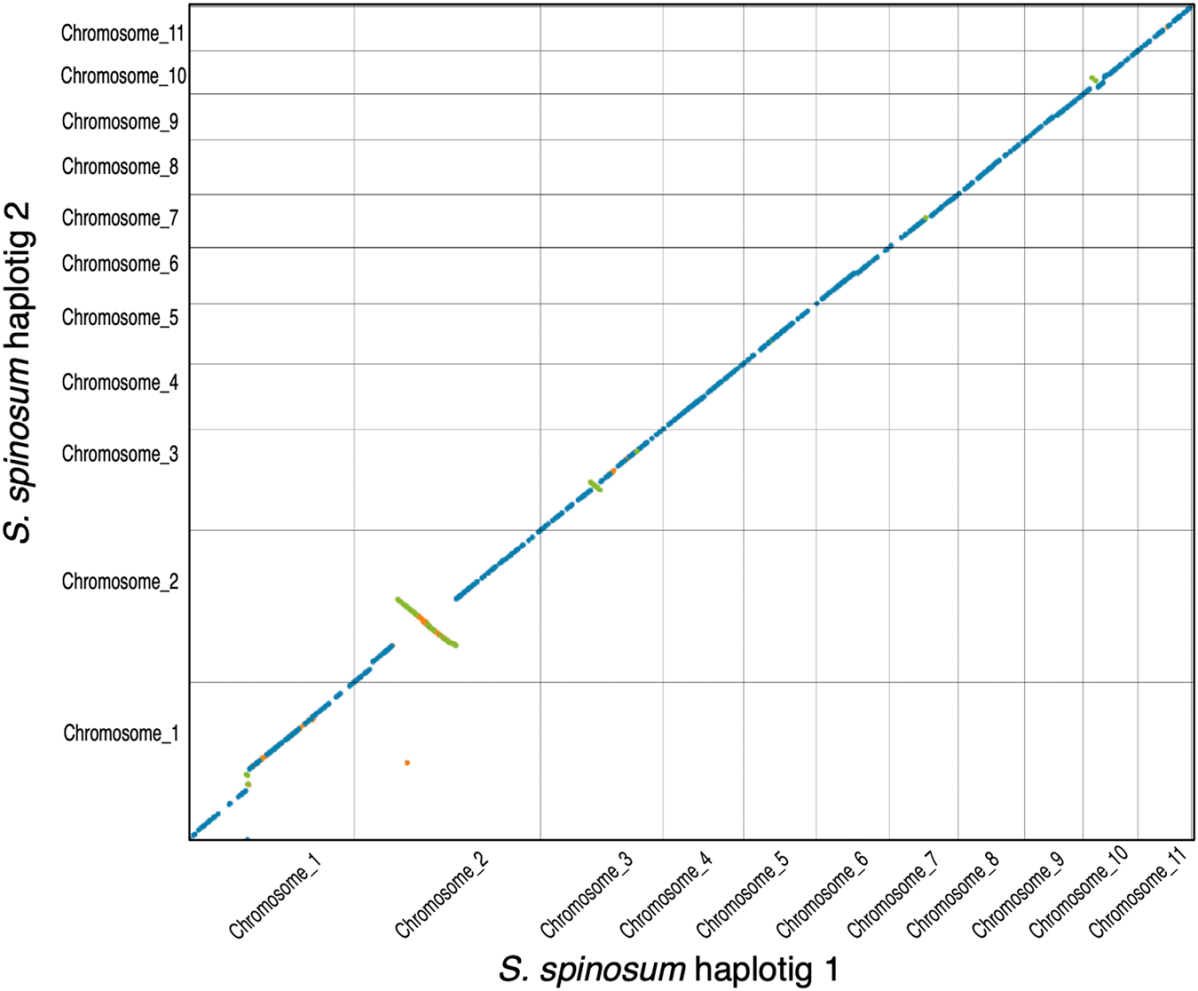
Collinearity between haplotig 1 and haplotig 2 of *S. spinosum*. Collinearity is shown as a dot plot representation along the eleven chromosomes. All chromosomes were highly collinear, except for chromosome 2 that exhibited an inversion.

We employed Nucmer^18^ and dot (https://github.com/marianattestad/dot) to compare the two parental argan genomes (haplotig 1 and haplotig 2). This comparison revealed extensive synteny and collinearity, with the exception of an inversion on chromosome 2 (Fig. 2). Taken together, these analyses confirm the genome structure of 11 chromosomes, as independently validated by the two parental genomes. Most chromosomes contained a strong telomere signal at both ends, with few exceptions (Fig. S2).

**Fig. 3:**
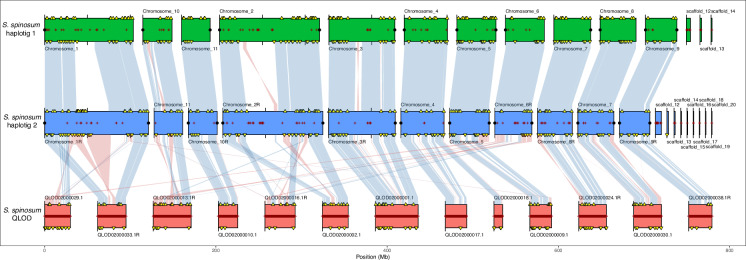
Synteny between different argan genome assemblies. Haplotig 1 (green) and haplotig 2 (blue) generated in this study were tested for synteny with the previously published genome assembly QLOD2 (orange). Red crosses along chromosomes represent assembly gaps, while black points at the end of chromosomes indicate telomere sequences. Yellow triangles indicate duplicated BUSCO genes in haplotig 1 and haplotig 2. Blue links between assemblies represent collinear regions, while red links between assemblies represent genome inversions.

We used Chromsyn^19^ to assess synteny between the two haplotigs and a previously published genome assembly of the species (QLOD00000000.2 from PRJNA294096)^3^. Overall, we observed strong synteny between haplotig 1 and haplotig 2, with only a few inversions near the centromeric regions of the larger chromosomes (Fig. 3). We also observed that the largest chromosomes (chromosome 1 and chromosome 2) were fragmented in the previously released Argan assembly (QLOD) (Fig. 3).

The result from the Chromsyn tool reflects an absence of synteny in the centromeric regions of chromosomes 1 and 2 between the two haplotigs. While this could be due to differences in transposable element content, a more plausible explanation is the limitation of the Chromsyn tool itself. Chromsyn relies on BUSCO orthologs to identify syntenic blocks^20^ which may not be effective in regions with extended repetitive sequences, and with very few coding sequences. The lack of annotated genes in these regions likely prevents the detection of synteny signals. However, the analysis of collinearity between haplotig 1 and haplotig 2 shows high homology at these regions of chromosomes 1 and 2 (Fig. 2). In general, evaluation and comparison of the genome assemblies with Quast^21^ revealed that all assembly statistics were improved in our two haplotigs relative to the QLOD assembly (Table S1), indicating that our new assembly represents a significant upgrade.

### Inversion in chromosome 2

We performed a detailed analysis of the inversion on chromosome 2. We used SAMTools depth^22^ to identify read coverage in regions flanking the inversion breakpoints. The boundaries of the inversion were identified through pairwise alignment of haplotypes (hap1 and hap2) using D-Genies^23^, with coordinates for hap1 defined as follows: start at 27.153 Mb, end at 63.831 Mb, and resumption of collinear sequence at 63.847 Mb (Fig. S3a).

Coverage analysis revealed a depth of 0 at the start of the inversion in hap1, indicating an absence of mapped reads at this breakpoint. This lack of coverage was visually confirmed with IGView^24^, hence, precluding to determine the exact location and nature of the beginning of the inversion (Fig. S3b). However, we observed overlapping reads at the end of the inversion and at the beginning of the downstream collinear region (Fig. S3b,c) confirming the presence of the inversion. In conclusion, while no overlapping reads are observed at the beginning of the inversion, the presence of overlapping reads at the end supports the existence of the structural variant, although its exact size remains uncertain.

**Fig. 4:**
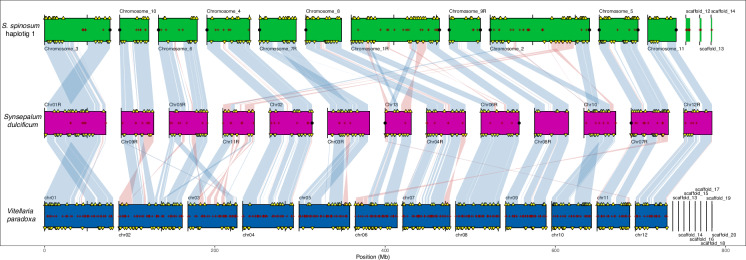
Synteny between three Sapotaceae species. The chromosomal complement of *S. spinosum* (green), *S. dulcificum* (magenta), and *V. paradoxa* (blue) were aligned. For clarity, only the *V. paradoxa* contigs exceeding 1 M bp are shown. Red crosses along the chromosomes represent assembly gaps, while black points at the ends of chromosomes represent telomere sequences. Yellow triangles indicate duplicated BUSCO genes. Blue links between assemblies represent collinear regions. Red links between assemblies represent genome inversions.

**Fig. 5:**
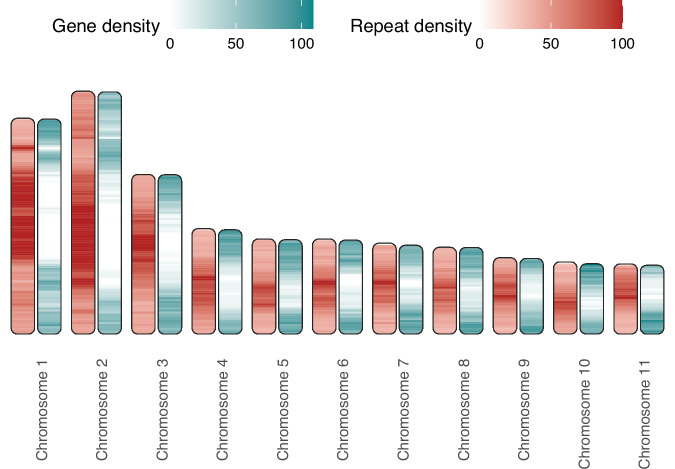
Gene and repeat density on haplotig 1. Density of predicted protein-coding genes (green) and repetitive sequences (red) are indicated by colour density gradients along the chromosomes of haplotig 1 (compare with Fig. S5).

The comparison with two other published genomes from the Sapotaceae family revealed similar chromosome numbers. The shea tree (*V. paradoxa*) contains 12 chromosomes^25^ and the miracle fruit tree (*S. dulcificum*) contains 13 chromosomes^26^. We used OMA 2.6.0^27^ to build a phylogenetic species tree using an orthologous matrix (OMA) based on 11291 orthologous groups of coding genes. Based on this tree, the shea tree is slightly closer to argan than to the miracle fruit tree (Fig. S4). The three Sapotaceae genomes exhibited extensive synteny and collinearity along their chromosomes, despite multiple chromosomal rearrangements and inversions (Fig. 4). Interestingly, the larger chromosomes in argan (chromosome 1 and chromosome 2) appear to have emerged as a result of repeat expansions and chromosome fusions (Figs. 4, 5): Argan chromosome 1 corresponds to shea tree chromosomes 6 and 8, and argan chromosome 2 corresponds to shea tree chromosomes 3 and 10.

### Repeat prediction

Repeat elements (including transposable elements) were predicted with RepeatModeler v2.0.1^28^, followed by soft-masking the genome assemblies with RepeatMasker v4.1.2^29^. We found that 60.86% of the hap1 and 60.78% of the hap2 genome consisted of repetitive regions (Table S2).

### Genome annotation

Genome annotation was performed with BRAKER3^30^, integrating both proteomic and RNA-seq data. Six different proteomes (*Medicago truncatula, V. paradoxa, S. dulcificum, Diospyros oleifera*, *Camellia sinensis* and *Rhododendron henanense*) were used as proteomic templates for gene prediction. RNA sequencing data were generated from three Argan root samples (PRJEB60382). These three RNA-seq libraries were initially subjected to quality filtering and trimming using fastp. The processed reads were subsequently mapped to the genome assembly with HISAT2^31^. This annotation pipeline resulted in the identification of 28,726 predicted genes for hap1 and 28,712 for hap2. A subsequent functional annotation was carried out using eggNOG^32^.

A global overview of gene and repeat density along all chromosomes shows a lack of genes and expansion of repetitive elements in the centromeric regions of the chromosomes on haplotig 1 (Fig. 5) and haplotig 2 (Fig. S5). Additionally, the two longest chromosomes in both parental genomes (hap1 and hap2) displayed large expansions of repetitive sequences, each encompassing more than 30 Mb (Fig. 5, S5).

### Chloroplast genome assembly

An independent approach was used to assemble the chloroplast genome. First, the HiFi reads were mapped to the published chloroplast reference genome of *S. spinosum* (MK533159.1)^6^ with minimap2^33^. The mapped reads were then recovered with SAMTools^22^ and the chloroplast genome was *de novo* assembled from the recovered reads using flye v.2.9.5^34^ with parameters–meta–pacbio -g 150k. We then used Bandage v.0.8.1^35^ to identify sequences that display circularity features. Finally, the assembly was annotated and visualized with the Proksee online tool^36^, which included Prokka annotation^37^. Collinearity between our assembly and published assemblies were evaluated with D-genies^23^.

**Fig. 6:**
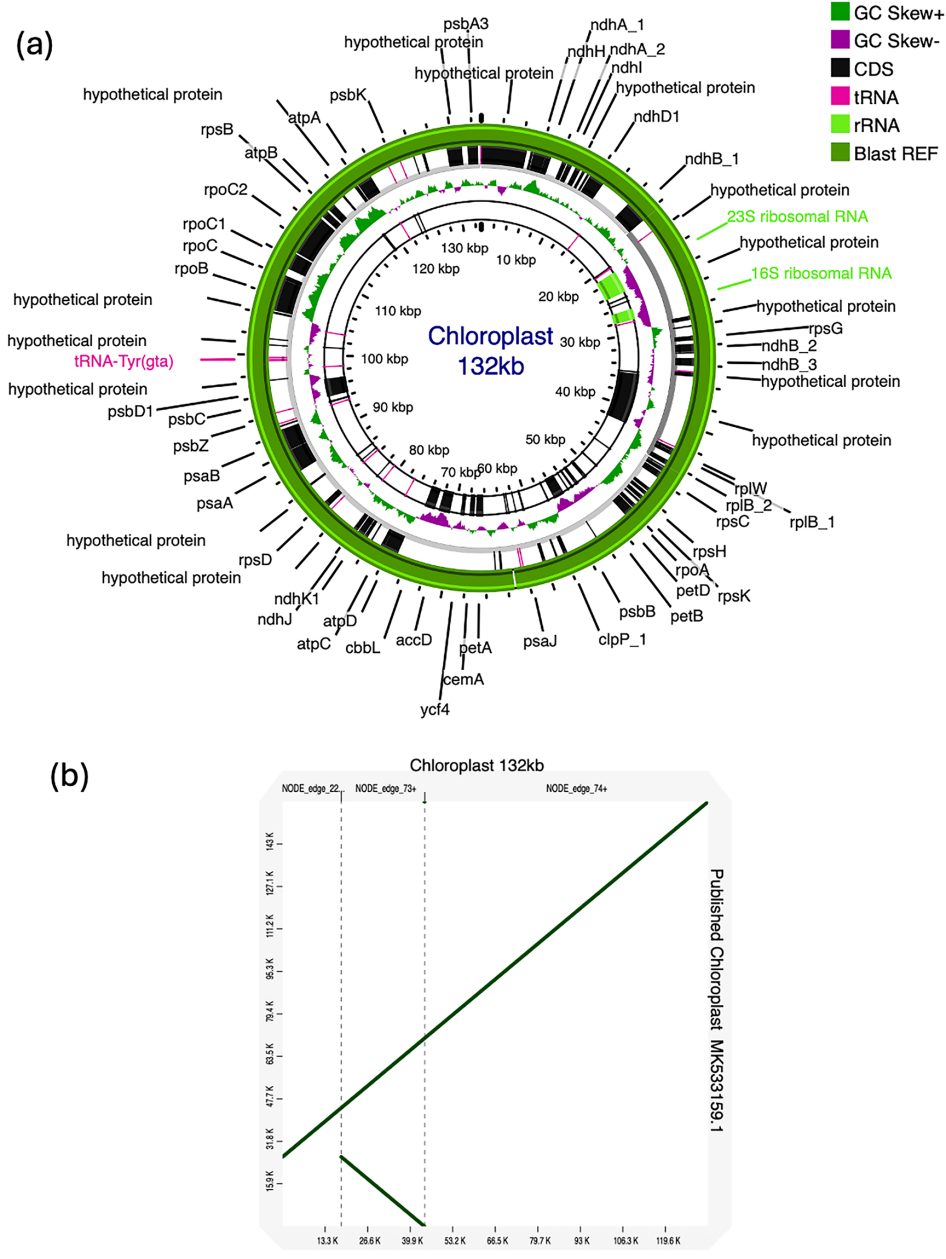
Chloroplast *de novo* assembly. (**a**) Annotated circular visualization of the assembled chloroplast genome. (**b**) Dot-plot representation of the collinearity between the newly assembled chloroplast genome and a previously published version of the chloroplast genome.

The chloroplast sequence consisted of 132,913 bp assembled from 3 contigs with 131 annotated features (Fig. 6a). Our new chloroplast assembly was blasted to a previously published chloroplast reference genome resulting in complete blast homology with excellent collinearity. The only difference with the published chloroplast sequence was a 26 kb duplication on the reference chloroplast assembly (Fig. 6b).

## Data Records

The three PacBio HiFi runs, and the Illumina Hi-C data can be accessed at the ENA repository https://www.ebi.ac.uk/ena/browser/view/PRJEB59883^38^ using the identification numbers ERR10968128, ERR10969898, ERR13030747, and ERR13030748, respectively. The genome assembly results are available in the ENA repository https://www.ebi.ac.uk/ena/browser/view/PRJEB88017 (hap1)^39^ and https://www.ebi.ac.uk/ena/browser/view/PRJEB88018^40^ (hap2). The genome annotations are available on Zenodo (https://zenodo.org/records/15913223)^41^.

## Technical Validation

Genome completeness was assessed using BUSCO v5.4.2^42^ with the eudicots_odb10 plant database, which contains 2,326 BUSCO entries. For hap1 and hap2 we found 97.8% and 98.5%, respectively, of the single-copy BUSCO entries (Tables S3, S4).

## Supplementary information

Supplementary Methods, Figures, and Tables

## Data Availability

The genome assembly scripts are available at https://github.com/ivandamg/Argan_Genome. All commands and pipelines used in data processing were executed according to the manual and protocols of the corresponding bioinformatic software.
