# Supplementary material for: Chromosome-level phased genome assembly of the argan tree Sideroxylon spinosum
Source: Sci Data. 2025 Aug 15;12:1430. doi: 10.1038/s41597-025-05768-1 (PMC12356838; doi:10.1038/s41597-025-05768-1)
Supplement: Supplementary file 1 — Supplementary Methods, Figures, and Tables [file 41597_2025_5768_MOESM1_ESM.pdf]

Chromosome-level phased genome assembly of the Argan tree *Sideroxylon spinosum*

Ivan D. Mateus[1], Abdellatif Essahibi[1], Pamela Nicholson[2], Mohamed Hijri[3], Ahmed Qaddoury[4], Laurent Falquet[1*], Didier Reinhardt[1*]

[1] Department of Biology, University of Fribourg, Chemin du Musée 10, 1700 Fribourg, Switzerland.

[2] Next Generation Sequencing Platform, University of Bern, Bremgartenstrasse 109a, 3012 Bern, Switzerland

[3] Department of Biological Sciences, University of Montréal, Québec, Canada

[4] Department of Biology, Faculty of Sciences and Techniques, University of Cadi Ayyad, Marrakesh, Morocco

[*] Corresponding authors:
Didier Reinhardt (didier.reinhardt@unifr.ch), Laurent Falquet (laurent.falquet@unifr.ch)

Supplementary Information

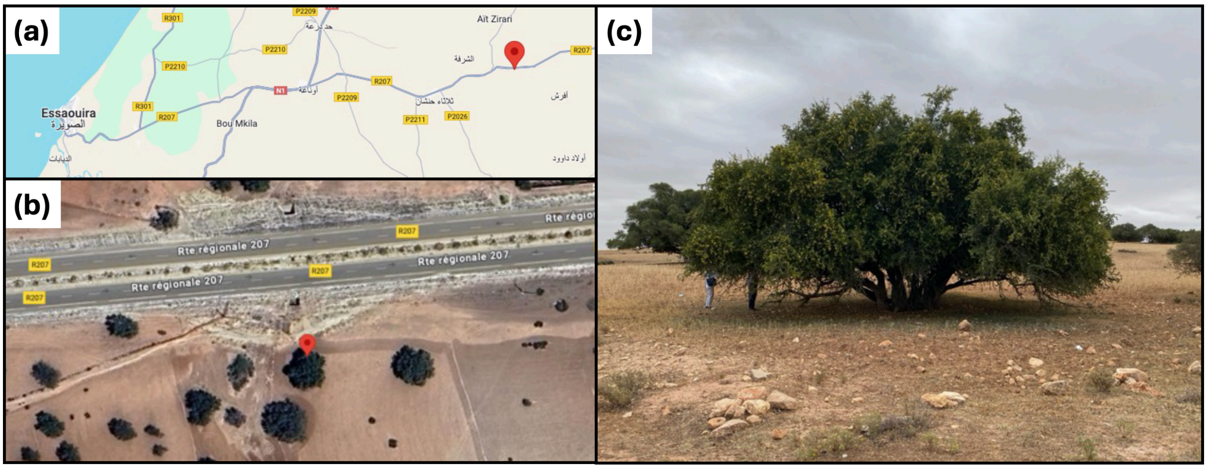

Figure S1. **Location of the argan tree sampled for genome sequencing.**
(a) and **(b)** show the exact location of the argan tree that was sampled for genome sequencing (coordinates 31°32'47.9" N 9°21'56.6" W). **(c)** Photograph of the sampled tree.

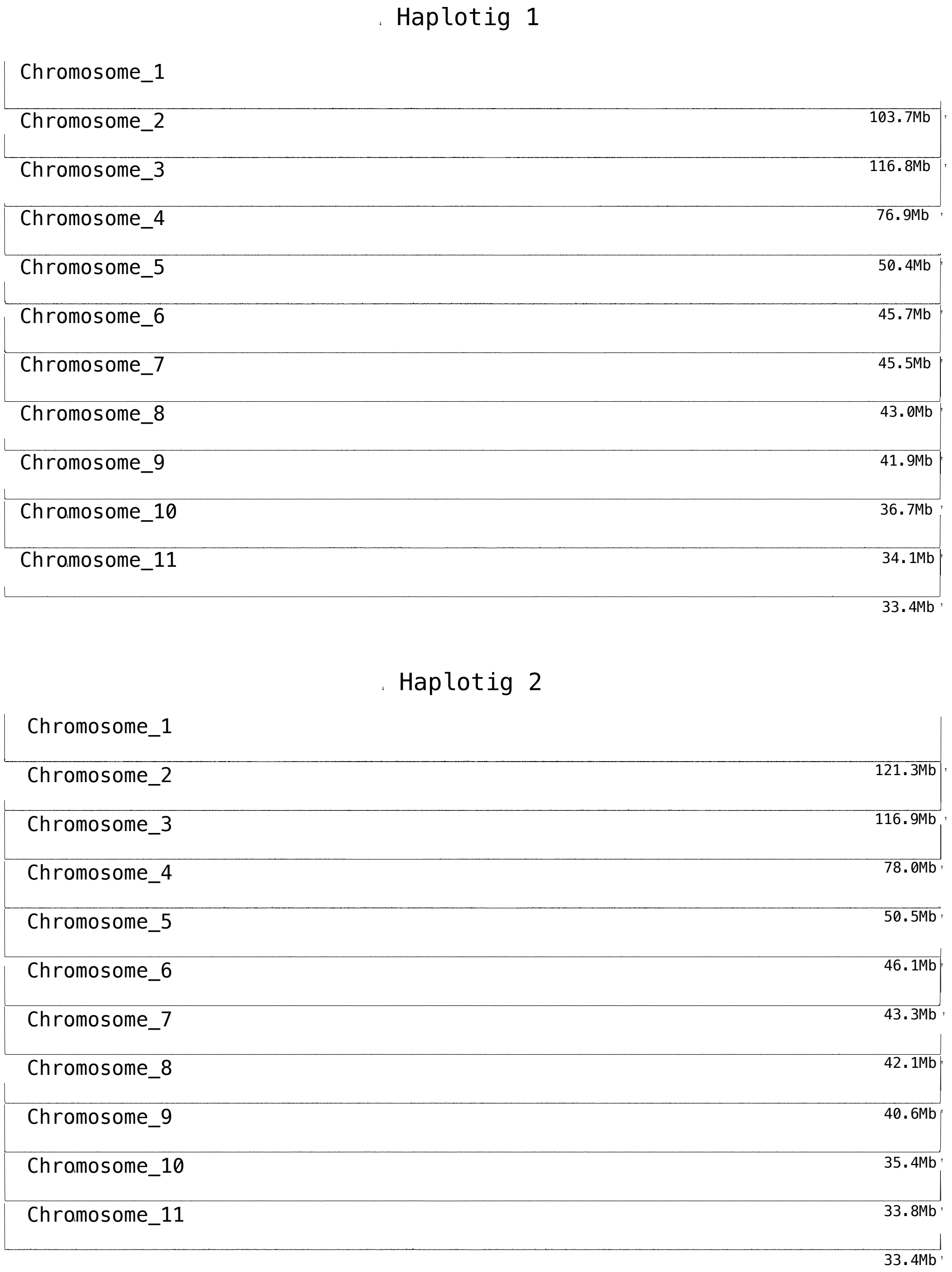

Figure S2. **Telomere analysis of the complete chromosomal set.**
Tidk plot representing the telomeres (vertical lines at the left and right end of chromosomes) of haplotig 1 and haplotig 2 at the 5' and 3' ends of the chromosomes (horizontal lines).

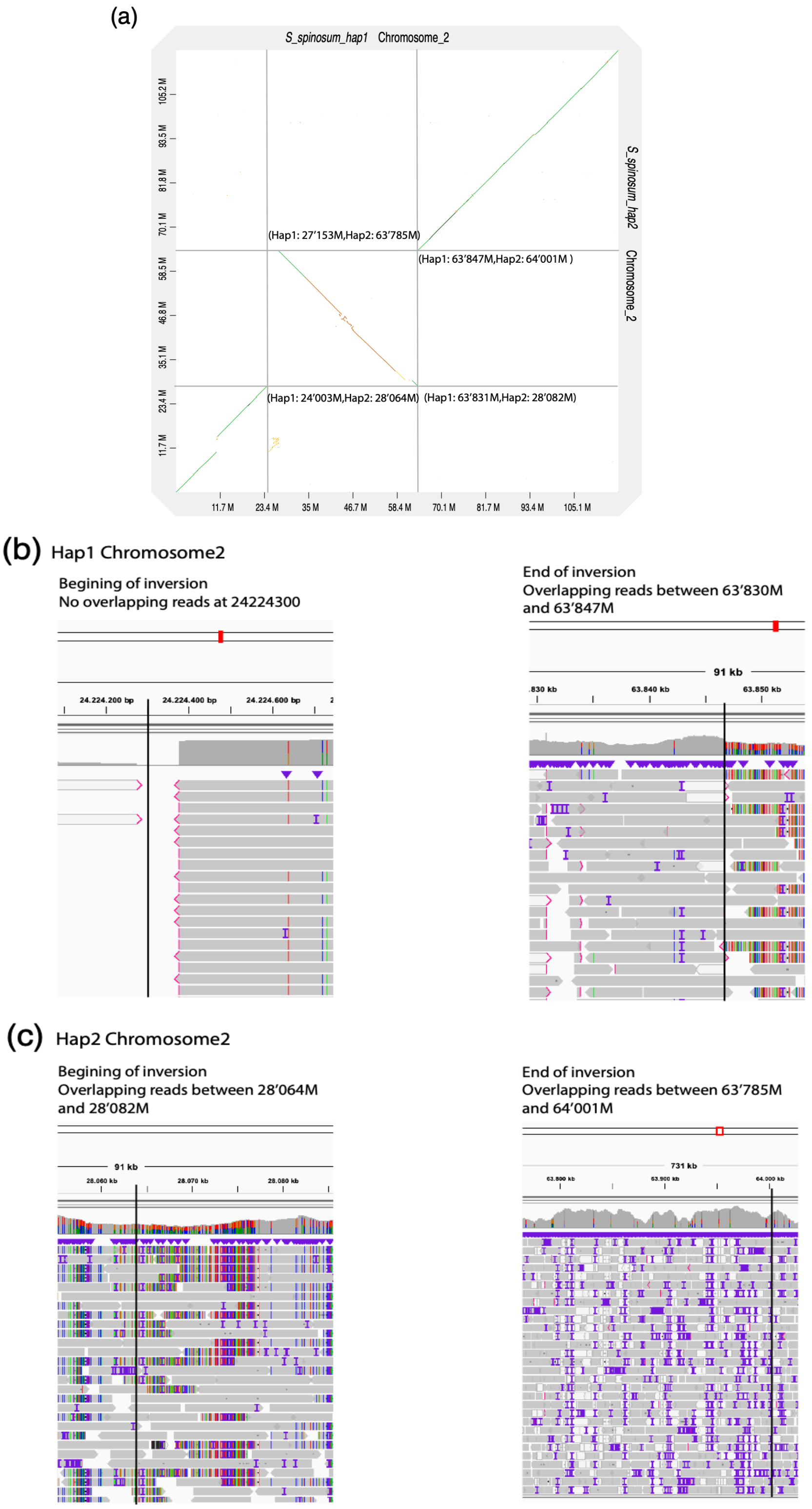

Figure S3. Analysis of the inversion in chromosome 2.
(**a**) Dot-plot representation of chromosome 2 comparing haplotig 1 and haplotig 2 shows collinearity and the breakpoints of the inversion in chromosome 2 between hap1 and hap2.
(**b**) IGView screenshots showing the coverage and mapped reads at the beginning and end of the inversion on haplotig 1. (**c**) IGView screenshots showing the coverage and mapped reads at the beginning and end of the region of haplotig 2 that corresponds to the inversion in haplotig 1. Vertical black lines in (**b**) and (**c**) indicate the positions of the breakpoints.

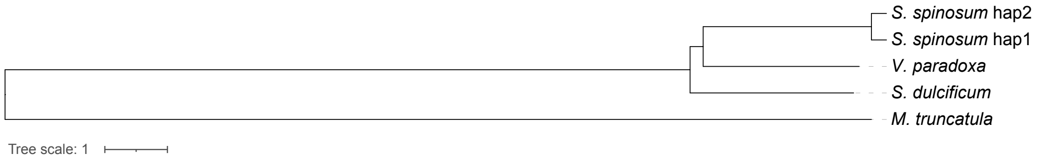

Figure S4. Phylogenetic analysis of three Sapotaceae genomes.
The two argan haplotigs were compared with the shea tree (*V. paradoxa*) and the miracle fruit tree (*S. dulcificum*) using *Medicago truncatula* as an outgroup. Phylogenetic analysis was based on 11291 OMA orthologous groups.

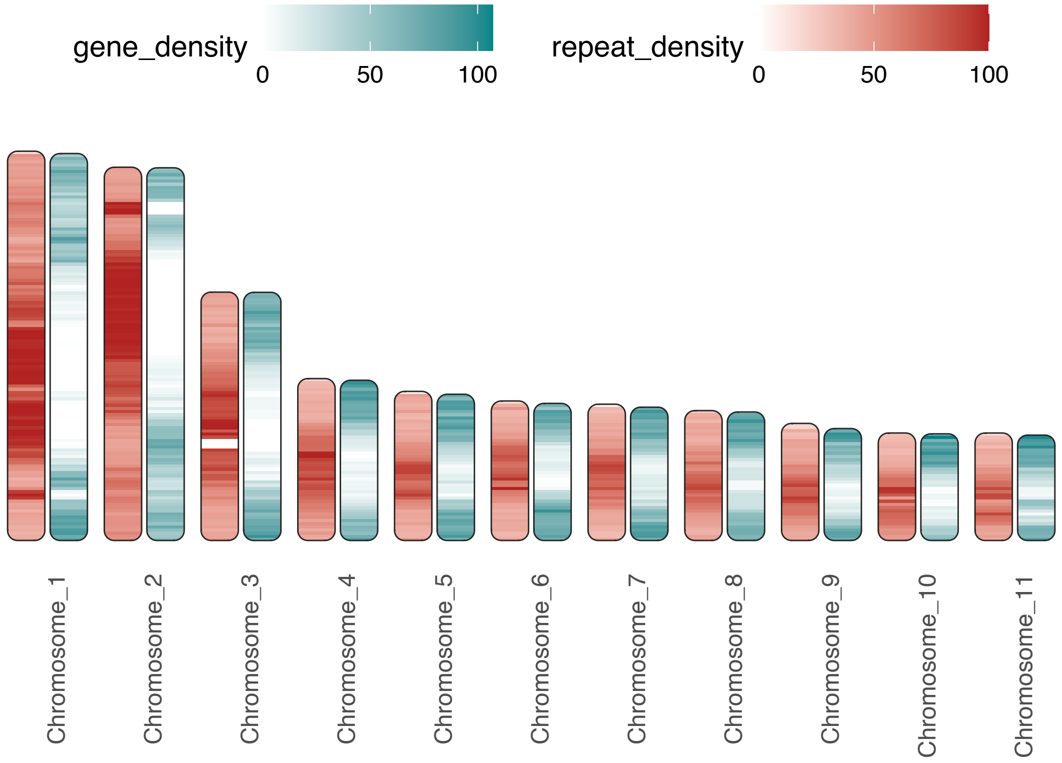

Figure S5. Gene and repeat density on haplotig 2.
Density of predicted protein-coding genes (green) and repetitive sequences (red) are indicated by colour density gradients along the eleven chromosomes of haplotig 2 (compare with **Fig. 5**).

Supplementary Methods

Isolation of high-molecular-weight genomic DNA for PacBio long-read sequencing

High-quality genomic DNA from argan (*Sideroxylon spinosum*) was isolated based on a modified CTAB protocol:

Solutions:

Extraction buffer (EB):
0.35 M sorbitol
100 mM Tris-HCl pH=7.5
10 mM EDTA

Lysis Buffer (LB):
200 mM Tris-HCl pH=7.5
50 mM EDTA
2M NaCl
2% w/v CTAB (Cetyltrimethylammonium bromide)

10% Sodium-N-lauroylsarcosinate

Procedure:

- Grind ca 5g of frozen argan leaves to finest possible powder in mortar with N2liq.
- Resuspend in 30 ml EB in 50ml Falcon tube
- Spin at 10'000 rpm for 20 min in Falcon swingout rotor
- remove supernatant (SN)
- resuspend pellet in 5 ml lysis buffer; move VERY GENTLY with a sterile spatule
- Transfer to 15 ml Falcon tube and add 1% sodium-N-lauroylsarcosinate
- incubate at 65°C for 2h in water bath
- after cooling, add 2.5 ml chloroform:isoamylalcohol (=3-methyl-1-butanol) 15:1
- mix VERY GENTLY by mild rocking for several minutes
- Spin at 10'000 rpm for 30 min in Falcon swingout rotor
- Collect SN; avoid ANY traces of the interface (better to sacrifice part of the DNA!)
- add equal volume of isopropanol and mix VERY GENTLY. Whitish precipitate forms within minutes at room temperature. Move solution VERY GENTLY by slow rocking until precipitate forms a cloud
- transfer gDNA "cloud" with toothpick to a new 15 ml Falcon tube and dissolve in 3 ml of LB
- incubate at 65°C for 2h in water bath
- add 2 ml chloroform:isoamylalcohol 15:1
- mix VERY GENTLY by mild rocking for several minutes
- Spin at 10'000 rpm for 30 min in Falcon swingout rotor
- Collect SN in a new 15 ml Falcon tube; avoid ANY traces of the interface or lower phase
- add equal volume of isopropanol and mix VERY GENTLY by slow rocking
- after 30 min precipitation at RT collect gDNA (10'000 rpm for 20 min)
- wash pellet with 70% ethanol for 30 min; dry pellet for several hours
- Dissolve DNA in water / TE

Isolation of nuclei for Hi-C sequencing

Nuclei from argan (*Sideroxylon spinosum*) were isolated using the CelLyticTM Plant Nuclei Extraction Kit (Sigma™ Cat # CELLYTPN1) according to the protocol of the provider with minor modifications:

Final Protocol:

- Grind 4g leaves in N2liq.
- Suspend in 20 ml 1x NIB (+1 mM DTT).
- Filter through nylon mesh.
- Spin down at 1260 g for 10 min.
- Resuspend pellet in 20 ml 1x NIB.
- Add 1 % Triton-X-100, shake well.
- Spin down at 12 g (300 rpm) 10 min.
- Collect SN and shake; spin down at 50 g (600 rpm) 30 min.
- Resuspend pellet in 7 ml NIB; add 1% Triton-X-100.
- Load onto gradient with 3 ml 2.3M sucrose / 3 ml 60% Percoll (**Figure S3**).
- Spin at 3000 rpm (1270 g) for 30 min.
- Collect Percoll phase (turbid; light green).
- Dilute with 1x NIB and collect at 1500 rpm (320 g) for 30 min.
- Wash with 1 ml NIB.
- Spin at 11000 g for 10 min and suspend in 100 µL PURE nuclei buffer.
- snap freeze in N2

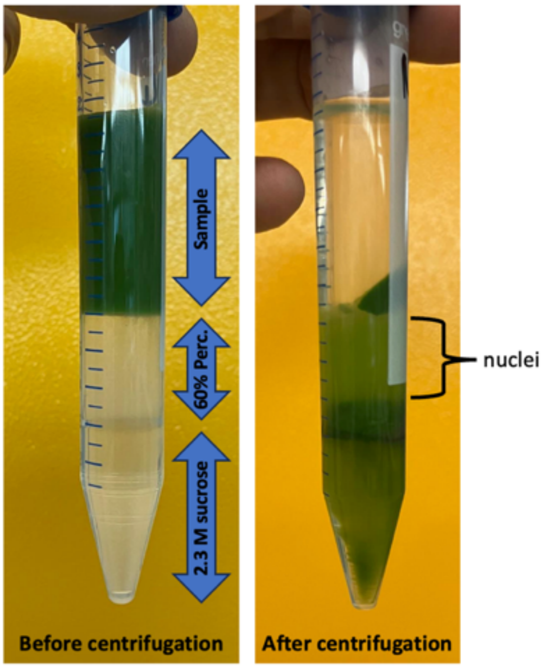

Figure S3. Isolation of argan nuclei by sucrose/Percoll gradient centrifugation

NIB: Nuclei Isolation Buffer

High-molecular-weight DNA quality check, library preparation, and PacBio sequencing

Prior to library preparation, high-molecular-weight genomic DNA was assessed for quantity, quality and purity using a Qubit 4.0 fluorometer (Qubit dsDNA HS or BR Assay kit Q32851 or Q32850, Thermo Fisher Scientific), an Advanced Analytical FEMTO Pulse instrument (Genomic DNA 165 kb Kit; FP-1002-0275, Agilent) and a Denovix DS-11 UV-Vis spectrophotometer, respectively. DNA recovered from plant nuclei was also used to prepare a PacBio low input SMRTbell library following the PacBio checklist entitled: *Procedure & Checklist - Preparing HiFi Libraries from Low DNA Input Using SMRTbell Express Template Prep Kit 2.0* (PacBio, PN 101-730-400 Version 07). Twenty ng of the nuclei-derived plant DNA was used to make SMRTbell libraries according to the guideline: "*Procedure & Checklist*

- *Preparing HiFi SMRTbell Libraries from Ultra-Low DNA Input*" PN 101-987-800 Version 02 (Nov 2021).
The CTAB-extracted high-molecular-weight genomic DNA was used to make a standard PacBio HiFi SMRTbell library according to the *Preparing whole genome and metagenome libraries using SMRTbell prep kit 3.0 Procedure & checklist* (PacBio, PN 102-166-600 REV01). Briefly, DNA sheared by Megaruptor 3 (speed 30/31) was concentrated and purified using 1 x SMRTbell clean-up beads. The samples were then quantified and qualified to be in the range of 15-18 Kb using a Qubit 4.0 fluorometer (Qubit dsDNA HS Assay kit; Q32851, Thermo Fisher Scientific) and an Advanced Analytical FEMTO Pulse instrument (Genomic DNA 165 kb Kit; FP-1002-0275, Agilent), respectively. The rest of the procedure as referenced above was followed including end-repair & A-tailing, ligation of barcoded overhang adapters and then purification of the library using AMPure PB beads as well as a nuclease treatment. All final libraries were checked for quantity and quality using a Qubit 4.0 fluorometer (Qubit dsDNA HS Assay kit; Q32851, Thermo Fisher Scientific) and an Advanced Analytical FEMTO Pulse instrument (Genomic DNA 165 kb Kit; FP-1002-0275, Agilent). Instructions in SMRT Link Sample Setup were followed to prepare the SMRTbell library for sequencing (PacBio SMRT Link v10 or 11). Shortly, PacBio Sequencing primer v3.2 and Sequel DNA Polymerase 3.0 were annealed and bound, respectively, to the DNA template libraries using a Sequel II Binding Kit 3.2 (PacBio Part number 102-333-300) and the complex was purified using SMRTbell clean-up beads. The libraries were loaded at an on-plate concentration of 50-150pM using adaptive loading, along with the use of Sequel II DNA internal control complex. SMRT sequencing was performed on the Sequel IIe with Sequel Sequencing kit 3.0, SMRT Cells 8M, 2 h pre-extension and a 30 h movie time. Thereafter, the CCS generation was performed on the Sequel IIe instrument and the barcode demultiplexing workflow was run in SMRT Link v11. All steps from gDNA extraction to data generation and utility were performed at the Next Generation Sequencing Platform, University of Bern, Switzerland.

DNA processing, library preparation, and sequencing for Hi-C analysis

High-molecular-weight genomic DNA from plant nuclei was prepared according to the "Isolating nuclei from plant tissue using LN2 disruption" Procedure & checklist (PacBio, PN 102-574-800, Rev01), followed by the "Nanobind HMW DNA extraction – plant nuclei" Procedure & checklist (PacBio, PN 102-574-200, Rev01). The Hi-C library was generated using an Arima Genomics Hi-C High Coverage Kit and following their user guide entitled Arima High Coverage HiC Kit (Arima Genomics document: A160667, initial release version). In brief, thawed nuclei were used for crosslinking and library preparation as described in the manual. Various quantification steps throughout the protocol and the final library were evaluated using a Thermo Fisher Scientific Qubit 4.0 fluorometer with the Qubit dsDNA HS Assay Kit (Thermo Fisher Scientific, Q32854) and an Agilent Fragment Analyzer (Agilent) with a HS NGS Fragment Kit (Agilent, DNF-474), respectively. The library was sequenced paired-end using an illumina NovaSeq 6000 S1 Reagent Kit v1.5 (300 cycles; illumina, 20028319) on an illumina NovaSeq 6000 instrument. The quality of the sequencing run was assessed using illumina Sequencing Analysis Viewer (illumina version 2.4.7) and all base call files were demultiplexed and converted into FASTQ files using illumina bcl2fastq conversion software v2.20. The quality control assessments, generation of libraries and sequencing was carried out at the Next Generation Sequencing Platform, University of Bern.

Assembly	*S._spinosum*_hap1	*S._spinosum*_hap2	*S._spinosum*_QLOD
# contigs (>= 0 bp)	14	20	186325
# contigs (>= 1000 bp)	14	20	79637
# contigs (>= 5000 bp)	14	20	8274
# contigs (>= 10000 bp)	14	20	2327
# contigs (>= 25000 bp)	14	20	268
# contigs (>= 50000 bp)	14	20	56
Total length (>= 0 bp)	636089469	654955621	690465237
Total length (>= 1000 bp)	636089469	654955621	617251183
Total length (>= 5000 bp)	636089469	654955621	478269350
Total length (>= 10000 bp)	636089469	654955621	438002149
Total length (>= 25000 bp)	636089469	654955621	408219428
Total length (>= 50000 bp)	636089469	654955621	401281704
# contigs	14	20	186325
Largest contig	116750276	121307699	49920449
Total length	636089469	654955621	690465237
GC (%)	34.09	34.18	32.57
N50	50434390	50469191	24953461
N75	43041221	42094784	3014
L50	4	4	11
L75	7	7	18670
# N's per 100 kbp	1.48	2.02	11831.95

Table S1. Quast report of genome assemblies. The *S. spinosum* haplotig 1 and haplotig 2 assemblies were compared with a previous genome assembly of the species.

	S. spinosum haplotig 1			*S. spinosum* haplotig 2		
sequences	14			20		
total length	63608946 bp			654955621 bp		
GC level	34.09%			34.18%		
bases masked	398384631 bp (62.63 %)			404898276 bp (61.82 %)		
	number of elements*	length occupied	percentage of sequence	number of elements*	length occupied	percentage of sequence
Retroelements	198079	154269473 bp	24.25%	196941	159838735 bp	24.40%
SINEs:	11075	1582809 bp	0.25%	6250	2922277 bp	0.45%
Penelope:	1620	354349 bp	0.06%	542	200823 bp	0.03%
LINEs:	47537	31611296 bp	4.97%	45128	25415231 bp	3.88%
CRE/SLACS	0	0 bp	0.00%	0	0 bp	0.00%
L2/CR1/Rex	0	0 bp	0.00%	0	0 bp	0.00%
R1/LOA/Jockey	74	84399 bp	0.01%	605	102268 bp	0.02%
R2/R4/NeSL	0	0 bp	0.00%	0	0 bp	0.00%
RTE/Bov-B	14374	8503015 bp	1.34%	13451	8435139 bp	1.29%
L1/CIN4	25897	16294489 bp	2.56%	24235	14806276 bp	2.26%
LTR elements:	137847	120721019 bp	18.98%	145021	131300404 bp	20.05%
BEL/Pao	1952	344844 bp	0.05%	116	40209 bp	0.01%
Ty1/Copia	45741	47193634 bp	7.42%	46084	47782107 bp	7.30%
Gypsy/DIRS1	52322	56896218 bp	8.94%	46424	53791999 bp	8.21%
Retroviral	2202	520755 bp	0.08%	2148	415671 bp	0.06%
DNA transposons	48486	18189792 bp	2.86%	37194	17378305 bp	2.65%
hobo-Activator	10701	5696725 bp	0.90%	10314	5358807 bp	0.82%
Tc1-IS630-Pogo	1106	546537 bp	0.09%	3028	1018803 bp	0.16%
En-Spm	0	0 bp	0.00%	0	0 bp	0.00%
MULE-MuDR	10146	5443525 bp	0.86%	4722	4043693 bp	0.62%
PiggyBac	0	0 bp	0.00%	0	0 bp	0.00%
Tourist/Harbinger	5671	1529072 bp	0.24%	2351	949529 bp	0.14%
Other (Mirage, P-element, Transib)	4688	701404 bp	0.11%	0	0 bp	0.00%
Rolling-circles	1848	1275553 bp	0.20%	3004	1396644 bp	0.21%
Unclassified:	615026	209454570 bp	32.93%	628986	210550985 bp	32.15%
Total interspersed repeats		381913835 bp	60.04%		387768025 bp	59.21%
Small RNA:	11988	6400808 bp	1.01%	7447	5968618 bp	0.91%
Satellites:	177	26404 bp	0.00%	2537	843040 bp	0.13%
Simple repeats:	238065	8394200 bp	1.32%	238722	8399224 bp	1.28%
Low complexity:	39289	1859318 bp	0.29%	39893	1897445 bp	0.29%

Table S2. RepeatMasker report.

S. spinosum haplotig 1.<br>Results from dataset eudicots_odb10	
C:97.8%[S:92.1%,D:5.7%],F:0.4%,M:1.8%,n:2326	
Complete BUSCOs (C)	2274
Complete and single-copy BUSCOs (S)	2142
Complete and duplicated BUSCOs (D)	132
Fragmented BUSCOs (F)	9
Missing BUSCOs (M)	43
Total BUSCO groups searched	2326

Table S3. Busco output on *S. spinosum* haplotig 1 from dataset eudicots_odb10 containing 2326 BUSCOs.

S. spinosum haplotig 2.<br>Results from dataset eudicots_odb10	
C:98.5%[S:92.6%,D:5.9%],F:0.3%,M:1.2%,n:2326	
Complete BUSCOs (C)	2291
Complete and single-copy BUSCOs (S)	2153
Complete and duplicated BUSCOs (D)	138
Fragmented BUSCOs (F)	7
Missing BUSCOs (M)	28
Total BUSCO groups searched	2326

Table S4. Busco output on *S. spinosum* haplotig 2 from dataset eudicots_odb10 containing 2326 BUSCOs.
